# Annexin A2 Is Associated with Dietary Cholesterol-Induced Metabolic Dysregulation and the Progression of Hepatic Fibrosis

**DOI:** 10.3390/metabo16050331

**Published:** 2026-05-15

**Authors:** Jiayang Liu, Ling Ou, Haiyan Tai, Yinghan Chai, Lirong Tan, Jie Lin, Bing Li, Ying Cao, Tingting Zhu

**Affiliations:** Guizhou University Medical College, Xiahui Road, Huaxi District, Guiyang 550025, China; liujiayang872@163.com (J.L.); oling1732@163.com (L.O.); m18230706045@163.com (H.T.); m18367077869@163.com (Y.C.); tanlirong1228@163.com (L.T.); linj@gzu.edu.cn (J.L.); bli23@gzu.edu.cn (B.L.)

**Keywords:** dietary cholesterol, liver, hepatic fibrosis, Annexin A2, hepatic lipotoxicity

## Abstract

**Background/Objectives**: Dietary cholesterol intake significantly influences liver health, yet the specific molecular mechanisms by which it accelerates fibrogenesis remain incompletely defined. This study aimed to characterize the dose-dependent effects of dietary cholesterol on hepatic injury and fibrogenesis, identify cholesterol-responsive gene networks through transcriptomic analysis, and investigate Annexin A2 (ANXA2) as a candidate molecular mediator linking dietary cholesterol to hepatic fibrosis progression. **Methods**: A CCl_4_-induced liver fibrosis mouse model was established and supplemented with dietary cholesterol (1–2%). Liver injury and fibrosis were assessed by liver-to-body weight ratios, serum biochemical markers, histological analysis, and fibrogenic gene expression. RNA sequencing combined with multiple hepatic fibrosis database analyses was performed to identify potential molecular mediators. **Results**: Dietary cholesterol supplementation aggravated CCl_4_-induced hepatic fibrosis in mice, with dose-dependent increases in liver-to-body weight ratios and serum AST and ALT levels. Histological analysis showed enhanced collagen deposition and upregulation of fibrogenic genes. By integrating RNA-sequencing with multiple hepatic fibrosis database analysis and correlation analysis, we identified Annexin A2 (ANXA2) as a cholesterol-responsive gene associated with fibrosis. **Conclusions**: Dietary cholesterol promotes liver fibrosis progression, and ANXA2 may act as a potential mediator linking cholesterol metabolism to hepatic fibrogenesis.

## 1. Introduction

Cholesterol is a fundamental nutrient found in everyday foods, which serve critical physiological roles in membrane integrity and hormone biosynthesis [1,2,3,4,5]. However, in the context of modern nutrition, excessive cholesterol intake is increasingly recognized as a functional trigger for metabolic dysfunction-associated steatotic liver disease (MASLD) [6,7,8]. Unlike general caloric excess, dietary cholesterol specifically exacerbates oxidative stress and pro-inflammatory signaling, creating a lipotoxic environment that promotes liver injury [9,10,11,12]. Therefore, it is increasingly important to examine how the cholesterol derived from everyday foods may trigger metabolic dysregulation and to identify the candidate molecular mediators that translate this nutritional exposure into liver pathophysiology.

Hepatic fibrosis is a critical manifestation of chronic liver injury, representing a key step in the progression from early fatty change or inflammation toward cirrhosis and liver-related morbidity [13]. It is characterized by activation of hepatic stellate cells (HSCs), excessive extracellular-matrix (ECM) deposition, disruption of normal liver architecture, and progressive loss of functional hepatocyte mass [14,15]. Beyond liver disease per se, hepatic fibrosis is associated with systemic complications including metabolic inflammation and extra-hepatic organ damage [16,17,18]. In clinical cohorts, the stage of liver fibrosis predicts progression to decompensation, hepatocellular carcinoma, and mortality much more reliably than steatosis or simple inflammation [19,20,21]. Against this backdrop, nutritional research increasingly considers the role of diet in fibrogenesis. Emerging work has directly implicated cholesterol overload in the liver as a driver of fibrosis: for instance, animal studies show that hepatic free-cholesterol accumulation in HSCs augments fibrogenic activation [22,23,24]. Despite evidence associating cholesterol accumulation with liver fibrosis, the specific impact of dietary cholesterol intake on fibrogenic signaling remains incompletely understood. Limited application of dose–response dietary models and integrative omics approaches has hindered the identification of nutrient-responsive molecular mediators. Consequently, the pathways through which dietary lipid exposure modulates fibrotic processes require further investigation.

Annexin A2 (ANXA2) represents a potential candidate for this mediating role. As a calcium-dependent phospholipid-binding protein, ANXA2 is uniquely positioned at the interface of lipid metabolism and cytoskeletal dynamics [25,26]. Moreover, ANXA2 has also been implicated in cholesterol and lipid homeostasis via modulation of LDL receptor degradation and membrane-associated lipid dynamics [27]. Recent studies show that ANXA2 expression is elevated in hepatic fibrosis and metabolic dysfunction-associated steatohepatitis (MASH) and promotes stellate-cell activation and fibrogenesis [28,29,30]. Based on these observations, we hypothesized that ANXA2 may be involved in the exacerbation of liver fibrosis in response to dietary cholesterol.

Consequently, this study aimed to characterize the dose-dependent metabolic and fibrogenic effects of dietary cholesterol, to identify cholesterol-responsive gene networks using transcriptomic analysis, and to investigate the association between ANXA2 expression and cholesterol-aggravated liver fibrosis. Collectively, these findings provide new insight into nutrient–gene interactions in diet-associated liver disease.

## 2. Materials and Methods

### 2.1. Experimental Diets, Animals and Design

CCl_4_-injected mice: Twenty C57BL/6 male mice were procured from Animal Tech Co. of SiPeiFu (Beijing, China) with an average age of 6–8 weeks old. After 1 week, the mice were randomly divided into 4 treatments with 5 replicates each. The first group was injected with corn oil (5 μL/g body weigh) as a mechanical control, and the other 3 groups received intraperitoneal injections of CCl_4_ (5 μL/g body weight, 10% in corn oil) twice weekly for 5 weeks. This model was selected because it reliably replicates the histopathological stages of human liver injury, including centrilobular necrosis and progressive collagen cross-linking [31].

The experimental treatment groups were as follows: (1) CON group: injected with corn oil as the vehicle control while being fed a standard diet and purified water; (2) CCl_4_ group: injected with CCl_4_ (using the method described previously) while being fed a standard diet and purified water; (3) 1% Chole. group: injected with CCl_4_ while being fed a diet containing 1% cholesterol and purified water; and (4) 2% Chole. group: injected with CCl_4_ (as previously described) while being fed a diet containing 2% cholesterol and purified water. The feeding regimen continued for 5 weeks, with body weight recorded twice weekly throughout the experimental period. This combination model was adopted based on established precedent [9,10], as dietary cholesterol alone requires extended feeding periods to induce significant fibrosis, whereas superimposing cholesterol supplementation on CCl_4_-induced injury allows efficient detection of cholesterol’s fibrosis-amplifying effects within a controlled timeframe. The detailed nutritional composition of the experimental diets is provided in Supplementary Table S1.

All the mice were reared in a specific pathogen-free (SPF) environment (temperature: 26 °C, 12 h light/dark cycle); after procurement and before the start of the experimental treatments, the mice were housed to acclimatize to the environment. All experimental procedures involving animals were approved by the Ethics Committee for Animal Use and Care of Guizhou University and complied with relevant ethical guidelines (Approval No. EAE-GZU-2025-T070, 10 March 2025).

### 2.2. Treatments and Sample Collection

CCl_4_-injected mice: Forty-eight hours after the final modeling injection, the mice were euthanized to collect blood and tissue samples. The intact liver was first excised and weighed. After photographic documentation, a portion was fixed in 10% formalin for histological examination, while the remaining tissue was flash-frozen in liquid nitrogen and stored at −80 °C for further analysis. Blood samples were allowed to stand at 4 °C for 4 h, followed by centrifugation (5000 rpm for 5 min at 4 °C) to isolate plasma. The resulting plasma samples were subsequently stored at −80 °C.

### 2.3. Biochemistry Analysis

Serum was obtained from the centrifuged blood samples as described above. Serum levels of alanine aminotransferase (ALT), aspartate aminotransferase (AST), triglycerides (TG), total cholesterol (TC) and low-density lipoprotein (LDL) were measured. The TG and TC levels in liver of mice were measured using commercially available kits (Nanjing Jiancheng Bioengineering Institute, Nanjing, China).

### 2.4. Histological Analysis

Fixed mouse liver tissues preserved in 10% formalin were dehydrated through a graded alcohol series, cleared in xylene, and then embedded in paraffin. The samples were sectioned into 4 μm thick slices using a microtome (Leica, Wetzlar, Germany). Tissue sections were stained with hematoxylin–eosin (H&E), Masson staining kit (Solarbio, Beijing, China) and Modified Sirius Red Stain Kit (Solarbio, Beijing, China). The processed sections were examined to evaluate hepatic pathological injury.

### 2.5. Cell Culture and Treatment

The study utilized LX-2 cells, which were kindly provided by Professor Bin Liang’s Laboratory at the Center for Life Sciences, Yunnan University, Yunnan, China. Cells were maintained in DMEM medium (Sangon Biotech, Shanghai, China) supplemented with 10% fetal bovine serum (Procell, Wuhan, China) and 1% penicillin–streptomycin (Yuanpei, Shanghai, China), and incubated in a humidified incubator at 37 °C and 5% CO_2_. TGF-β_1_ stimulation: LX-2 cells were treated with TGF-β_1_ (10 ng/mL, MCE, Monmouth Junction, NJ, USA) [32] for 24 h.

### 2.6. mRNA Expression Analysis

The total RNA of liver tissue and cells was isolated using RNAiso Plus (TaKaRa, Dalian, China) and RNA extraction assisted reagent (Accurate biology, Changsha, China). Subsequently, complementary DNA (cDNA) was synthesized using Evo Super M-MLV Reverse Transcriptase II (Accurate Biology, China) with 1000 ng of total RNA as template. The cDNA was amplified using the SYBR green qPCR mix (Accurate biology, China), and the primers were purchased from Sangon Biotech (China); the sequences are listed in Supplementary Table S3. The relative expressions of the target genes were determined by the 2^−ΔΔCt^ method using *Actb* as the control.

### 2.7. Transcriptome Analysis

LX-2 cells and liver tissues from three randomly selected mice from each group were used for RNA extraction. They were frozen in dry ice and mailed to a commercial company (Novogene Co., Ltd., Kunming, China). Total RNA was extracted and transcriptome sequencing was performed based on the Illumina sequencing platform. Raw reads were first quality-filtered using fastp (v0.19.7). The resulting clean reads were then aligned to the mouse reference genome (GRCm39/mm39) using HISAT2 (v2.0.5), and gene-level read counts were quantified using featureCounts (v1.5.0-p3). Differential gene expression analysis was performed using DESeq2 (v1.20.0) with Benjamini–Hochberg correction; differentially expressed genes (DEGs) were defined by |log_2_ fold change| ≥ 1 and adjusted *p* < 0.05. GO and KEGG enrichment analyses were performed using clusterProfiler (v3.8.1). Detailed RNA-seq library quality metrics and genome alignment statistics are provided in Supplementary Table S6. In addition, we also utilized the GEPIA2 (gene expression profiling interactive analysis-2) database. The sequencing data were analyzed using the NovoMagic (www.novogene.com), and Sangerbox (SangerBox.com).

### 2.8. Western Blot

Liver tissues and cells were lysed with RIPA buffer (high) (Solarbio, China) supplemented with PMSF (Solarbio, China) to obtain protein lysate. The slurry was centrifuged at 12,000 rpm at 4 °C for 15 min, and the supernatant was collected for protein quantification by the BCA method. Protein samples were separated on 12% SDS-polyacrylamide gel, imprinted onto PVDF membrane (Millipore Corp, Billerica, MA, USA), and blocked the membrane for 1 h, which was then incubated with primary antibodies for 12 h at 4 °C. Following washing, the membrane was incubated with the secondary antibody for 1 h. The antibodies were listed in Supplementary Table S5. These bands were detected and displayed using a chemiluminescent detection solution (Biosharp, Hefei, China) and an automated chemiluminescence analyzer (Tanon, Shanghai, China).

### 2.9. Statistical Analysis

All analyses were carried out in triplicate times. Statistical analyses were performed using one-way analysis of variance (ANOVA) or Student’s *t*-test. Spearman’s rank correlation analysis was applied to assess the relationship between gene expression levels. GraphPad Prism 9.0 was used for statistical analysis of the graphs, with significance thresholds set at *p* < 0.05, *p* < 0.01, and *p* < 0.001. Findings are displayed as mean ± standard error of the mean (SEM, indicated by error bars). The graphical abstract was created using BioRender (https://www.biorender.com/).

## 3. Results

### 3.1. Dysregulated Cholesterol Homeostasis Is Associated with Hepatic Fibrosis

**Figure 1:**
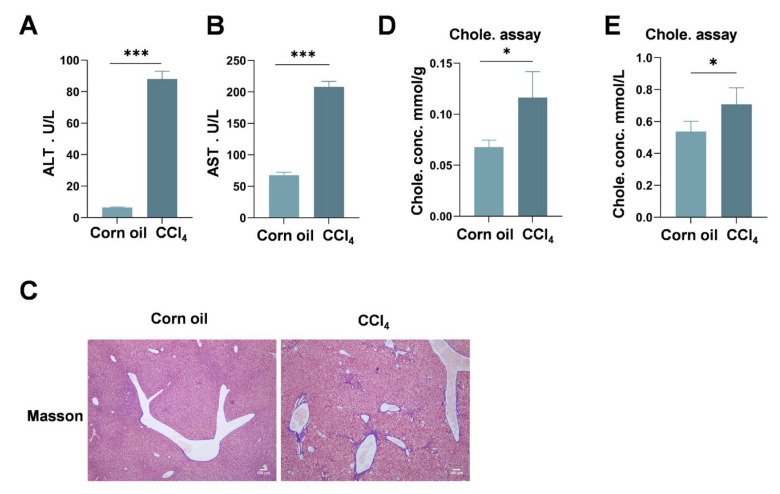
**Cholesterol is significantly increased upon liver fibrosis in MAFLD patients and mice.** (**A**) Serum levels of ALT. (**B**) Serum levels of AST. (**C**) Masson staining of mouse liver histological sections. (**D**) Liver tissue total cholesterol concentration and serum total cholesterol (**E**) concentration of CCl_4_-treated mice and control mice. Statistical significance was analyzed using a two-tailed Student’s *t* test ((**A**,**B**,**D**,**E**), * *p* < 0.05, *** *p* < 0.001).

To investigate the relationship between cholesterol metabolism and hepatic fibrosis, we first established a CCl_4_-induced mouse model of liver fibrosis. CCl_4_ treatment led to significant elevations in serum ALT and AST levels (Figure 1A,B) and markedly increased collagen deposition in liver tissues (Figure 1C). Notably, both serum and hepatic cholesterol levels were significantly elevated in CCl_4_-treated mice compared with controls (Figure 1D,E), indicating a strong association between cholesterol accumulation and fibrotic progression. Consistently, a survey of published clinical studies further supported this trend (Table 1).

Building upon these findings, we next examined cholesterol metabolism at the gene-expression level. In the CCl_4_-induced mouse fibrosis model, qRT-PCR analysis showed significant downregulation of key cholesterol synthesis, transport, and clearance genes, including *Apom*, *Lss*, *Ldlr*, *Apoe*, *Hmgcr*, *Sqle* and *Srebf2*—relative to controls (Figure S1A,B). This suppression pattern was further validated in vitro using a TGF-β1-induced LX-2 hepatic stellate cell model, where activated HSCs displayed robust induction of mesenchymal markers accompanied by a parallel decrease in cholesterol metabolism-related genes (Figure S1C,D). Together, these in vivo, in vitro, and clinical findings indicate that cholesterol accumulation coincides with a broad suppression of cholesterol metabolic pathways, supporting a critical regulatory role for disrupted cholesterol homeostasis in the development and progression of hepatic fibrosis.

### 3.2. Cholesterol Biosynthesis Was Inhibited in Hepatic Fibrosis

**Figure 2:**
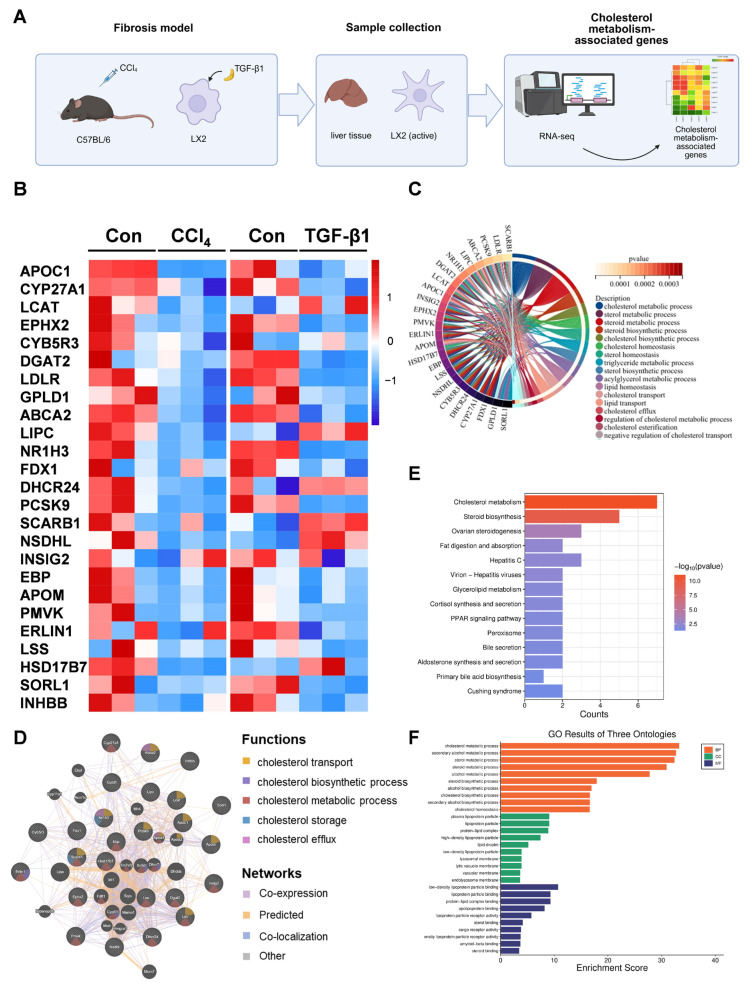
**RNA-Seq Profiling Identifies Cholesterol Metabolic Dysregulation in Liver Fibrosis.** (**A**) Schematic of fibrosis liver tissue and LX-2 cell activation model. (**B**) Heatmap of the down-regulated genes involved in cholesterol metabolism upon control and CCl_4_-induced liver injury mice, and with/without 24 h TGF-β1 treatment in LX-2 cells. Number of independent experiments [*n*] = 3. (**C**) The chord diagram illustrates the main biological processes of the decreased genes. (**D**) Networking interpretation of the down-regulated genes. (**E**,**F**) KEGG/GO enrichment analysis of the inhibited cholesterol metabolic genes. KEGG, Kyoto Encyclopedia of Genes and Genomes; GO, Gene Ontology.

These observations prompted a comprehensive investigation into the transcriptional landscape of cholesterol metabolism to elucidate the underlying dysregulation during fibrogenesis. We performed RNA sequencing on TGF-β1-stimulated LX-2 cells and CCl_4_-induced fibrotic liver tissues, along with their respective controls (Figure 2A). Comparative transcriptomic analysis consistently demonstrated significant downregulation of cholesterol metabolism genes in both models, visualized through cross-species heatmaps (Figure 2B), a trend that aligned with patterns observed in publicly available GSE datasets (Figure S2). The chord diagram indicated that these down-regulated genes are related to cholesterol metabolic process, cholesterol biosynthetic process, cholesterol homeostasis and cholesterol transport (Figure 2C), which supported the relationship of liver fibrosis and cholesterol metabolism. In addition, network analysis suggested that the majority of these genes display co-expression and colocalization (Figure 2D). Kyoto Encyclopedia of Genes and Genomes (KEGG) and Gene Ontology (GO) enrichment analysis also showed that these down-regulated genes are involved in cholesterol metabolism process, cholesterol biosynthetic process and cholesterol homeostasis (Figure 2E,F).

### 3.3. Dietary Cholesterol Exacerbates CCl4-Induced Hepatic Fibrosis in Mice

**Figure 3:**
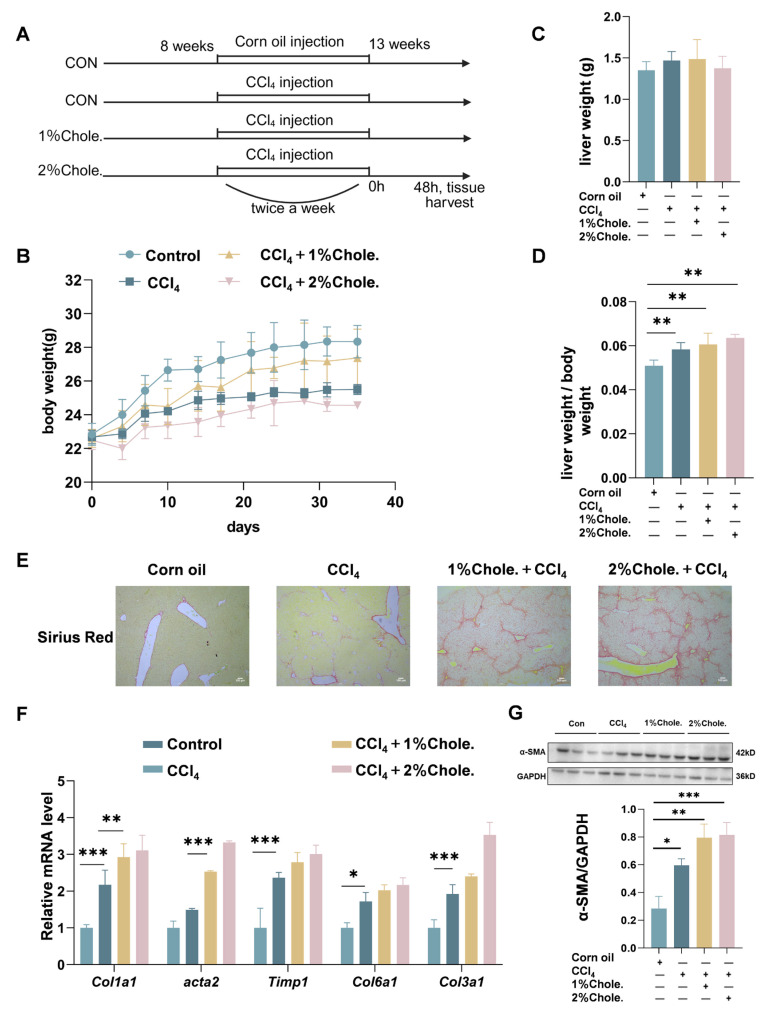
**Cholesterol promotes the progression of hepatic fibrosis in mice.** (**A**) Experimental flowchart of the experimental procedure used to examine the adverse effects of cholesterol in liver fibrosis mice for 5 weeks. Liver fibrosis mice were intraperitoneally injected corn oil or CCl_4_ (5 μL/g body weight, 10% in corn oil) twice a week beginning at week 8 for 5 weeks. [*n*] = 5 per group. (**B**) Body weight of mice. (**C**) Liver weight of mice. (**D**) LW/BW value of mice. (**E**) Sirius Red staining of mouse liver histological sections. (**F**) Quantitative PCR was performed to determine the hepatic mRNA levels of genes related to fibrosis markers (*Col1a1*, *Acta2*, *Timp1*, *Col6a1*, *Col3a1*) in mice from the indicated groups. Gene expression was normalized to *Actb* mRNA levels. [*n*] = 3 per group. (**G**) Expression of α-SMA and glyceraldehyde 3-phosphate dehydrogenase (GAPDH) was analyzed by Western blot. GAPDH served as a loading control. [*n*] = 3 per group. The data were analyzed with one-way ANOVA, * *p* < 0.05, ** *p* < 0.01, *** *p* < 0.001; Scale bar, 100 μm; CCl_4_, carbon tetrachloride; Chole., cholesterol.

The above findings demonstrate that cholesterol levels are markedly elevated in liver fibrosis, despite the downregulation of genes involved in cholesterol metabolism. This paradox suggests that dietary and/or other exogenous sources of cholesterol may contribute significantly to the initiation and progression of liver fibrosis. To investigate the impact of dietary cholesterol on hepatic fibrosis, we employed a carbon tetrachloride (CCl_4_)-induced mouse model of hepatic injury, in which animals were fed cholesterol-supplemented diets at varying concentrations (Figure 3A). Throughout the experimental period, mice receiving a normal chow diet maintained significantly higher body weights compared to those on cholesterol-enriched diets (Figure 3B and Figure S3A). Mice administered cholesterol-supplemented diets exhibited aggravated hepatic fibrosis in response to CCl_4_, as evidenced by increased liver weight (LW) (Figure 3C) and elevated liver-to-body weight (LW/BW) ratios (Figure 3D). Sirius Red staining further revealed that dietary cholesterol potentiated CCl_4_-induced collagen deposition and fibrotic remodeling in the liver (Figure 3E). Consistently, qRT-PCR analysis showed significant upregulation of fibrosis-associated genes, including *Col1a1*, *Acta2*, *Timp1*, *Col6a1* and *Col3a1*, in cholesterol-fed mice (Figure 3F). At the protein level, expression of alpha-smooth muscle actin (α-SMA), a key marker of hepatic stellate cell activation, was also markedly increased in the presence of dietary cholesterol (Figure 3G). Collectively, these data indicate that exogenous cholesterol exacerbates hepatic injury and promotes fibrogenesis in the context of CCl_4_-induced hepatic fibrosis.

### 3.4. Dietary Cholesterol Amplifies Hepatic Inflammation and Hepatic Injury

**Figure 4:**
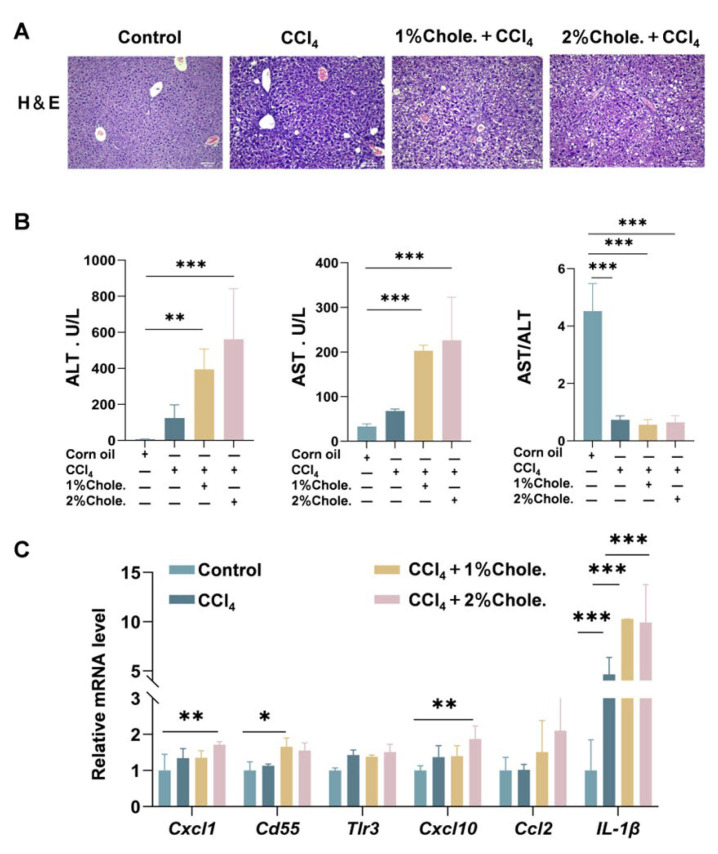
**Cholesterol feeding increases liver inflammation.** (**A**) H&E staining of mouse liver histological sections. (**B**) Serum levels of ALT, AST, AST/ALT. [*n*] = 3 per group. (**C**) Quantitative PCR was performed to determine the hepatic mRNA levels of genes related to inflammation markers (*Cxcl1*, *Cd55*, *Tlr3*, *Cxcl10*, *Ccl2*, *IL-1β*) in mice from the indicated groups. Gene expression was normalized to *Actb* mRNA levels. [*n*] = 3 per group. The data were analyzed with one-way ANOVA, * *p* < 0.05, ** *p* < 0.01, *** *p* < 0.001; Scale bar, 100 μm; AST, Aspartate Aminotransferase; ALT, Alanine Aminotransferase.

Building upon the observed exacerbation of hepatic fibrosis by dietary cholesterol, we further evaluated its effects on hepatitis inflammation and hepatic injury. Histopathological examination using H&E staining demonstrated significantly more severe inflammatory infiltration and necrotic foci in CCl_4_-treated mice, with pronounced aggravation in cholesterol-supplemented groups (Figure 4A). Consistently, biochemical assessment of serum markers revealed substantially elevated levels of ALT, AST, and AST/ALT ratio in cholesterol-fed mice, exhibiting a dose-dependent response (peak elevation in 2% cholesterol group; Figure 4B). Notably, the dose-dependent elevations in serum ALT and AST observed in mice fed 1% and 2% cholesterol (Figure 4B) are consistent with the positive associations between dietary cholesterol intake and histological disease severity reported in human MAFLD cohorts (Table 1). Furthermore, mice fed the cholesterol-supplemented diet exhibited a dose-dependent decrease in serum high-density lipoprotein (HDL) levels and an increase in low-density lipoprotein (LDL) levels (Figure S3B,C), confirming augmented hepatocellular damage. Molecular profiling via qRT-PCR analysis further showed significantly upregulated expression of key inflammatory mediators (*Cxcl1*, *Cd55*, *Tlr3*, *Cxcl10*, *Ccl2*, *Il-1β*) in cholesterol-supplemented mice compared to controls (Figure 4C). Collectively, the data suggest that dietary cholesterol plays a detrimental role in worsening hepatic injury by reinforcing both inflammatory and metabolic stress in the fibrotic liver.

### 3.5. Transcriptomic Profiling Unravels a Dose-Dependent Cholesterol-Driven Pro-Fibrotic Program

**Figure 5:**
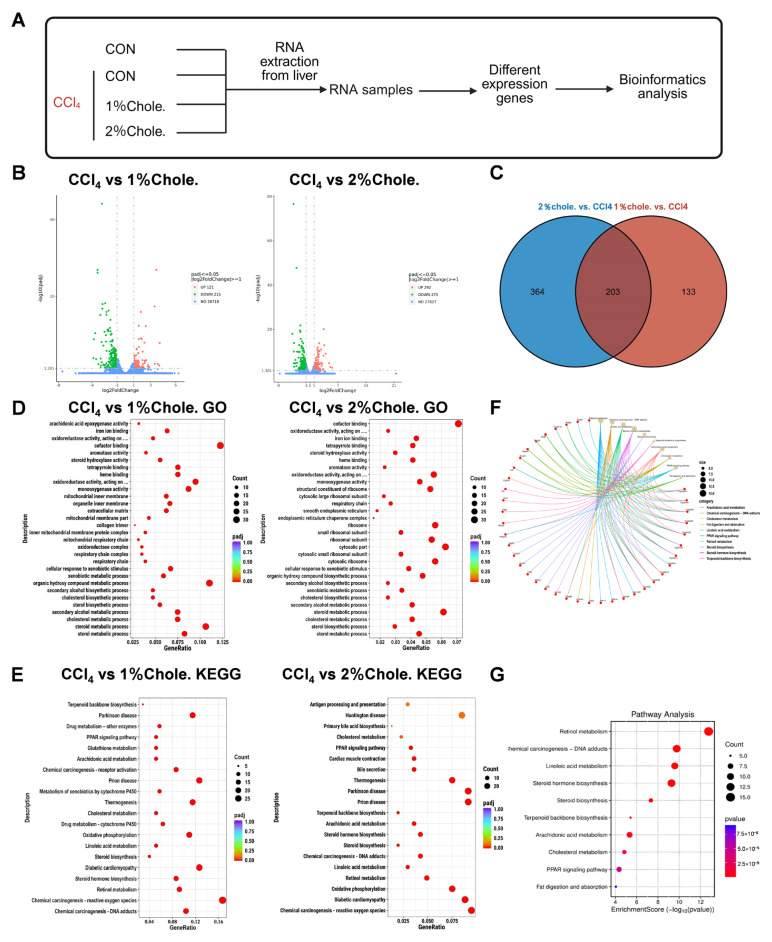
**Transcriptomic analysis reveals activation of pathways in cholesterol-fed mice.** (**A**) RNA-seq schematic flowchart of control and liver fibrosis mice. [*n*] = 3. (**B**) Volcano plots depicting differentially expressed genes (DEGs) in liver tissues of CCl_4_-induced fibrotic mice fed 1% or 2% cholesterol-supplemented diets compared with the CCl_4_-only group (|log_2_FC| > 1.5, adjusted *p* < 0.05); gray dots represent non-significant genes. (**C**) Venn diagram of liver tissue DEGs between CCl4 vs. 1%Chole. and CCl4 vs. 2%Chole. (**D**,**E**) Signaling pathway analysis of the differential genes in CCl4 vs. 1%Chole. and CCl4 vs. 2%Chole. (**F**) Chord diagram illustrates the main biological processes of the genes from Venn diagram. (**G**) Signaling pathway analysis of the genes from Venn diagram. In (**D**), truncated GO terms are displayed as ‘oxidoreductase activity, acting on....’. The full annotations are: (1) oxidoreductase activity, acting on paired donors, with incorporation or reduction of molecular oxygen; (2) oxidoreductase activity, acting on paired donors, with incorporation or reduction of molecular oxygen, reduced flavin or flavoprotein as one donor, and incorporation of one atom of oxygen.

To elucidate the molecular mechanisms by which dietary cholesterol aggravates liver fibrosis, we performed transcriptomic profiling (RNA-seq) on liver tissues from CCl_4_- or corn oil-treated mice fed with either normal chow or cholesterol-enriched diets (Figure 5A). Compared to the CCl_4_ group, the 1% cholesterol group exhibited 121 upregulated and 215 downregulated genes, while the 2% cholesterol group showed 292 upregulated and 275 downregulated genes (Figure 5B). Among these, 203 differentially expressed genes (DEGs) were commonly altered in both cholesterol-fed groups (Figure 5C and Supplementary Table S4), indicating consistent transcriptomic shifts in response to dietary cholesterol. Gene Ontology (GO) and Kyoto Encyclopedia of Genes and Genomes (KEGG) enrichment analyses of DEGs in both the 1% vs. CCl_4_ and 2% vs. CCl_4_ comparisons revealed significant enrichment in pathways related to cholesterol metabolism (Figure 5D,E). A chord diagram visualizing the 203 overlapping DEGs further confirmed their strong association with these pathways (Figure 5F). Subsequent KEGG enrichment of the 203 crossover DEGs revealed enrichment in cholesterol-related processes (Figure 5G). Collectively, these findings suggest that dietary cholesterol induces broad transcriptomic alterations in the liver, a process that ultimately promotes the development and progression of liver fibrosis.

### 3.6. ANXA2 Identified as a Consistently Upregulated Cholesterol-Associated Gene Implicated in Liver Fibrosis Progression

**Figure 6:**
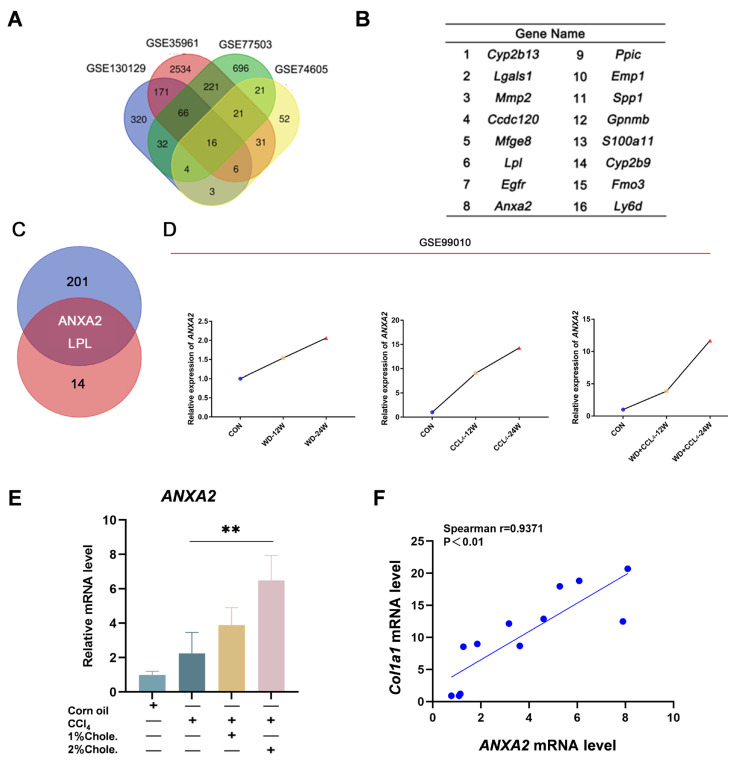
**ANXA2 is upregulated during the progression of liver fibrosis.** (**A**) Venn diagram displaying the intersection of differentially expressed genes (DEGs) across four transcriptomic datasets. (**B**) Cholesterol-responsive fibrosis-associated differentially expressed gene set. (**C**) Venn diagram shows the intersection between 16-gene set and cholesterol-associated fibrotic genes. (**D**) *ANXA2* gene expression across different groups in the GSE99010 dataset. (**E**) qRT-PCR was performed to determine the hepatic mRNA levels of *Anxa2* in mice from the indicated groups. Gene expression was normalized to *Actb* mRNA levels. [*n*] = 3 per group. (**F**) Correlation analysis between *Anxa2* and *Col1a1* mRNA expression levels in liver tissues. Spearman’s rank correlation coefficient was calculated to assess the association between *Anxa2* and *Col1a1* expression. The data were analyzed with one-way ANOVA, ** *p* < 0.01.

Through transcriptomic analysis, we identified 203 candidate genes potentially involved in the development of liver fibrosis. To further refine the core regulatory factors, we conducted an integrative analysis across four murine fibrosis models. Cross-comparison of transcriptomic datasets (GSE130129, GSE35961, GSE77503, and GSE74605, provided in Supplementary Table S2) revealed 16 genes consistently dysregulated in all models (Figure 6A,B). To identify genes that may participate specifically in cholesterol-aggravated fibrogenesis, we intersected this 16-gene set with our previously defined panel of 203 cholesterol-associated fibrotic genes, yielding only two overlapping candidates: Anxa2 and Lpl (Figure 6C). LPL primarily mediates triglyceride hydrolysis and has been mechanistically linked to VLDL secretion rather than fibrosis [36]. We focused our investigation on Anxa2, whose role in cholesterol-associated hepatic fibrosis remains incompletely understood.

Analysis of the publicly available GSE99010 dataset demonstrated that Anxa2 expression was similarly increased in both Western diet-induced and CCl_4_-induced liver injury, displaying a progressive rise with disease severity (Figure 6D). Moreover, qRT-PCR analysis revealed that ANXA2 expression was increased in the model group in a dose-dependent manner in response to dietary cholesterol, and Spearman correlation analysis showed a positive association between ANXA2 and *Col1a1* expression from the mouse model (Figure 6E,F). Furthermore, GEPIA2-based analysis demonstrated that ANXA2 expression was progressively upregulated from normal liver tissues to fibrosis, cirrhosis, and hepatocellular carcinoma (HCC) (Figure S4). Together, these findings identify ANXA2 as a consistently upregulated, cholesterol-associated gene that may play an important regulatory role in the progression of liver fibrosis.

## 4. Discussion

Emerging evidence highlights the significant impact of dietary lipids, particularly cholesterol, on liver pathology. Previous studies have firmly established that dietary cholesterol can exacerbate liver fibrosis [9]. In clinical and preclinical settings of MASLD, cholesterol overload has been linked to scar progression and fibrotic remodeling [20]. However, the transcriptional changes underlying this process have remained underexplored. In our study, we observed that fibrosis is associated with elevated serum and hepatic cholesterol, while key genes involved in cholesterol synthesis, transport, and clearance (e.g., *Apom*, *Lss*, *Ldlr*, *Apoe*, *Hmgcr*, and *Sqle*) are markedly downregulated. These findings add a new layer of mechanistic understanding by linking cholesterol accumulation not only to HSC activation, but to broad suppressive reprogramming of cholesterol-metabolic gene expression.

While cholesterol accumulation has been implicated in HSCs activation, few studies have performed genome-wide transcriptomics to chart how cholesterol metabolism is remodeled during fibrogenesis [23]. Using RNA-seq in both TGF-β_1_-stimulated LX-2 cells and CCl_4_-treated fibrotic livers, we uncovered a coordinated downregulation of cholesterol metabolism, biosynthesis, homeostasis, and transport pathways. Functional enrichment (GO, KEGG) analysis reinforced that these genes operate in a tightly regulated module that is disrupted in fibrosis. This transcriptomic reprogramming suggests that fibrotic livers may downregulate cholesterol biosynthesis even as cholesterol accumulates, possibly as a maladaptive response or feedback mechanism. The paradoxical coexistence of elevated hepatic cholesterol and suppressed endogenous cholesterol biosynthesis warrants mechanistic consideration. Under physiological conditions, intracellular cholesterol accumulation suppresses the proteolytic activation of sterol regulatory element-binding protein 2 (SREBP-2) at the endoplasmic reticulum, thereby downregulating its transcriptional targets through a classic negative feedback loop [37]. In the fibrotic liver, this feedback suppression may be further exacerbated by the fibrotic microenvironment itself. TGF-β1, a master regulator of HSC activation, has been shown to directly suppress SREBP-2 nuclear translocation and impair cholesterol biosynthetic gene expression in hepatic cells [23], suggesting that fibrogenic signaling and cholesterol sensing pathways are mechanistically intertwined. Simultaneously, the broad downregulation of cholesterol export and reverse transport genes observed in our data indicates that impaired cholesterol clearance and efflux may be equally, if not more, responsible for net hepatic cholesterol retention than biosynthetic suppression alone. Together, these observations suggest a maladaptive cycle in the fibrotic liver: dietary cholesterol overload overwhelms an already compromised clearance capacity, while the fibrotic microenvironment simultaneously suppresses endogenous synthesis through SREBP-2-dependent feedback, paradoxically amplifying cholesterol accumulation rather than resolving it. Elucidating the precise contribution of SREBP-2 dysregulation and HSC-mediated cholesterol sensing to this cycle represents an important direction for future mechanistic investigation.

Beyond the transcriptional mechanisms discussed above, it is also informative to contextualize our model within the broader landscape of preclinical MASLD research. Several preclinical models have been used to study MASLD-associated fibrosis, each with distinct strengths and limitations. The methionine- and choline-deficient (MCD) diet induces rapid fibrosis but causes weight loss and lacks the metabolic syndrome phenotype characteristic of human MASLD [31]. Western diet (WD) models more faithfully recapitulate human metabolic context but require 16–24 weeks to develop significant fibrosis [38]. Genetic models such as ob/ob and db/db mice develop severe steatosis but require a secondary hit to progress to fibrosis, and their monogenic etiology limits translational relevance [39]. The model employed in this study offers a practical advantage: superimposing cholesterol supplementation onto an established fibrotic background enables efficient detection of cholesterol’s fibrosis-amplifying effects within a shorter experimental window.

Dietary cholesterol amplifies fibrosis via metabolic and signaling networks. Prior work has shown that dietary cholesterol augments fibrosis in models like bile-duct ligation or CCl_4_ injury, largely through accumulating free cholesterol in HSCs and enhancing TLR4 signaling [40]. Our study extends these findings by profiling transcriptomic shifts induced by varying doses of dietary cholesterol in a CCl_4_ fibrosis model. We identified hundreds of differentially expressed genes (DEGs) common to both 1% and 2% cholesterol supplementation groups; notably, a core set of 203 genes was enriched for cholesterol metabolism and liver fibrosis. Through integrated intersection analysis of RNA-seq results and multiple publicly available databases, ANXA2 was identified as a candidate gene associated with cholesterol metabolism and liver fibrosis. These findings suggest that ANXA2 may serve as a candidate mediator linking dietary cholesterol overload to the progression of hepatic fibrosis, thereby providing a focused molecular target for further mechanistic investigation.

ANXA2 has been increasingly implicated in liver diseases. For instance, in metabolic dysfunction-associated steatohepatitis (MASH), a positive regulatory loop between ANXA2 and Notch signaling in hepatocytes was shown to promote fibrosis via osteopontin secretion [28]. Moreover, recent work has linked ANXA2 to metabolic stress in NAFLD: activation of TLR4 upregulates ANXA2 via NF-κB p65 and c-Jun, which impairs AMPK/mTOR-dependent autophagy and exacerbates lipid accumulation [41]. In alcoholic liver fibrosis, treatment with isoliquiritigenin suppressed ANXA2 and reversed fibrogenic activation, further supporting its role in HSCs’ biology [42]. Building on these observations, our integrative intersection analysis across four murine fibrosis datasets, a cholesterol-associated gene panel, and our RNA-seq results identified ANXA2 (alongside LPL) as a consistently overlapping candidate gene. We further validated that ANXA2 expression was markedly upregulated in cholesterol-fed fibrotic livers and in publicly available datasets. qRT-PCR analysis confirmed that ANXA2 was significantly increased in the model group and exhibited a dose-dependent response to dietary cholesterol. Additionally, Spearman correlation analysis demonstrated a positive association between ANXA2 and *Col1a1* expression in the mouse model. Moreover, analysis of GEPIA2 datasets revealed that ANXA2 expression was significantly elevated in tumor tissues of liver cancers, underscoring its potential clinical relevance.

Several limitations of this study should be noted. First, as the present findings are based on transcriptomic profiling and correlational analyses, the functional role of ANXA2 in cholesterol-driven fibrogenesis remains to be addressed in future work. Gain- or loss-of-function experiments in relevant cell models would help further elucidate the underlying mechanisms. Second, the present study was conducted exclusively in male C57BL/6 mice. While this strain is among the most widely used and well-characterized for liver disease research, whether the observed effects on cholesterol-driven fibrosis and ANXA2 expression apply equally to female animals or broader human populations remains to be determined. Given well-documented sex differences in hepatic lipid metabolism, estrogen-mediated cholesterol regulation, and susceptibility to liver fibrosis, validating the cholesterol–ANXA2 association in female models and across diverse human cohorts represents an important direction for future investigation. Third, while mRNA expression of ANXA2 was validated across experimental groups, protein-level quantification across all dietary cholesterol conditions and the CCl_4_ model warrants further consolidation in future studies. Finally, the CCl_4_ model reliably replicates key histopathological features of liver injury but does not fully capture the metabolic complexity of human MASLD; complementary diet-induced models would strengthen the translational relevance of these findings. In summary, this study reveals that dietary cholesterol exacerbates fibrosis through a dual mechanism: it accumulates in the liver while paradoxically suppressing endogenous synthesis. These findings provide mechanistic insight into how cholesterol accelerates liver fibrosis and highlight ANXA2 as a candidate molecular mediator warranting functional validation through gain- and loss-of-function studies in future work.

## Figures and Tables

**Table 1:** Dietary Cholesterol Intake and MAFLD in Humans: A Summary.

Population	Mean Age (Years)	BMI (kg/m^2^)	Cholesterol Consumption	Key Conclusion	References
50 patients 25 MASH, 25 controls ^b^	Control: 37 ± 9 NASH: 37 ± 10	Control: 24.9 ± 2.5 NASH: 25.6 ± 2.5	Control: 405 ± 111 NASH: 506 ± 108 *p* = 0.002 **	Dietary intake of MASH patients was richer in cholesterol.	Musso et al. 2003 [33]
73 MAFLD ^a^	Control: 46.8 ± 2.7 Steatosis: 44.7 ± 2.7 NASH: 47.7 ± 2.2	Control: 28.5 ± 1.4 Steatosis: 27.1 ± 1.1 NASH: 32.1 ± 0.8	Control: 269.5 ± 27.5 steatosis: 290.8 ± 28.1 MASH: 357.9 ± 37.5	Increased dietary intake correlated with histologic disease severity.	Allard et al. 2008 [34]
56 MAFLD ^a^	Non-obese:47.2 ± 14.8 Obese: 53.5 ± 12.3	Non-obese: <25 Obese: >25	*p* = 0.0378 *	Cholesterol intake was significantly higher in patients.	Yasutake et al. 2009 [20]
>215,000 MAFLD/Cirrhosis ^a^	MAFLD: 57.2 ± 7.8 Cirrhosis: 59.9 ± 7.5	MAFLD: 26.9 ± 4.9 Cirrhosis: 29.8 ± 6.0	MAFLD: 1.16 (*p* = 0.005 **) Cirrhosis:1.52 (*p* = 0.002 **)	Cholesterol intake positively associated with MAFLD with cirrhosis.	Noureddin et al. 2020 [19]
608 Cirrhosis	Not report	Not report	*p* = 0.004 **	Cholesterol was associated with a 46% increase in the risk of clinical or histologic progression.	Yu et al. 2013 [21]
9221 Survey ^b^	Control: 48.7 ± 15.7 Cirrhosis-Related or Liver Cancer-Related: 53.5 ± 13.7	Control: 25.7 ± 5.2 Cirrhosis-Related or Liver Cancer-Related: 26.9 ± 5.4	*p* = 0.007 **	Cholesterol positively associated with cirrhosis and liver cancer.	Ioannou et al. 2009 [35]

^a^: Results are reported as means ± SEM. ^b^: Results are reported as means ± SD. * *p* < 0.05, ** *p* < 0.01.

## Data Availability

The original contributions presented in this study are included in the article/Supplementary Materials. Further inquiries can be directed to the corresponding authors.

## Supplementary Materials

The following supporting information can be downloaded at: https://www.mdpi.com/article/10.3390/metabo16050331/s1, Figure S1: Expression of Cholesterol Metabolism-Related Genes during Liver Fibrosis; Figure S2: Heatmap of the genes involved in cholesterol metabolism in GEO database; Figure S3: Cholesterol promotes the progression of hepatic fibrosis in mice; Figure S4: ANXA2 mRNA expression is upregulated during liver disease progression; Table S1: Diet Disclosure (1–2% Cholesterol diet); Table S2: The RNA-seq databases information; Table S3: Primers for Real-Time PCR detection; Table S4: 203 cholesterol-associated fibrotic genes; Table S5: Antibodies; Table S6: Summary of RNA-seq library quality metrics and genome alignment statistics.

## Abbreviations

ANXA2, Annexin A2; ALT, alanine aminotransferase; AST, aspartate aminotransferase; CCl_4_, carbon tetrachloride; *Col1a1*, Collagen Iα1; DEGs, differentially expressed genes; GO, gene ontology; HDL, high-density lipoprotein; H&E, hematoxylin-eosin; HSCs, hepatic stellate cells; KEGG, Kyoto Encyclopedia of Genes and Genomes; LDL, low-density lipoprotein; MASH, metabolic dysfunction-associated steatohepatitis; MASLD, metabolic dysfunction-associated steatotic liver disease; MCD, methionine- and choline-deficient; PVDF, Polyvinylidene difluoride; qRT-PCR, quantitative real-time polymerase chain reaction; RNA-seq, RNA sequencing; SREBP-2, sterol regulatory element-binding protein 2; TC, total cholesterol; TG, triglycerides; TGF-β, transforming growth factor beta; WB, Western blot; WD, Western diet.
