# Supplementary material for: Annexin A2 Is Associated with Dietary Cholesterol-Induced Metabolic Dysregulation and the Progression of Hepatic Fibrosis
Source: Metabolites. 2026 May 15;16(5):331. doi: 10.3390/metabo16050331 (PMC13208583; doi:10.3390/metabo16050331)
Supplement: Supplementary file 1 [file metabolites-16-00331-s001.zip › metabolites-4288476-supplementary.pdf]

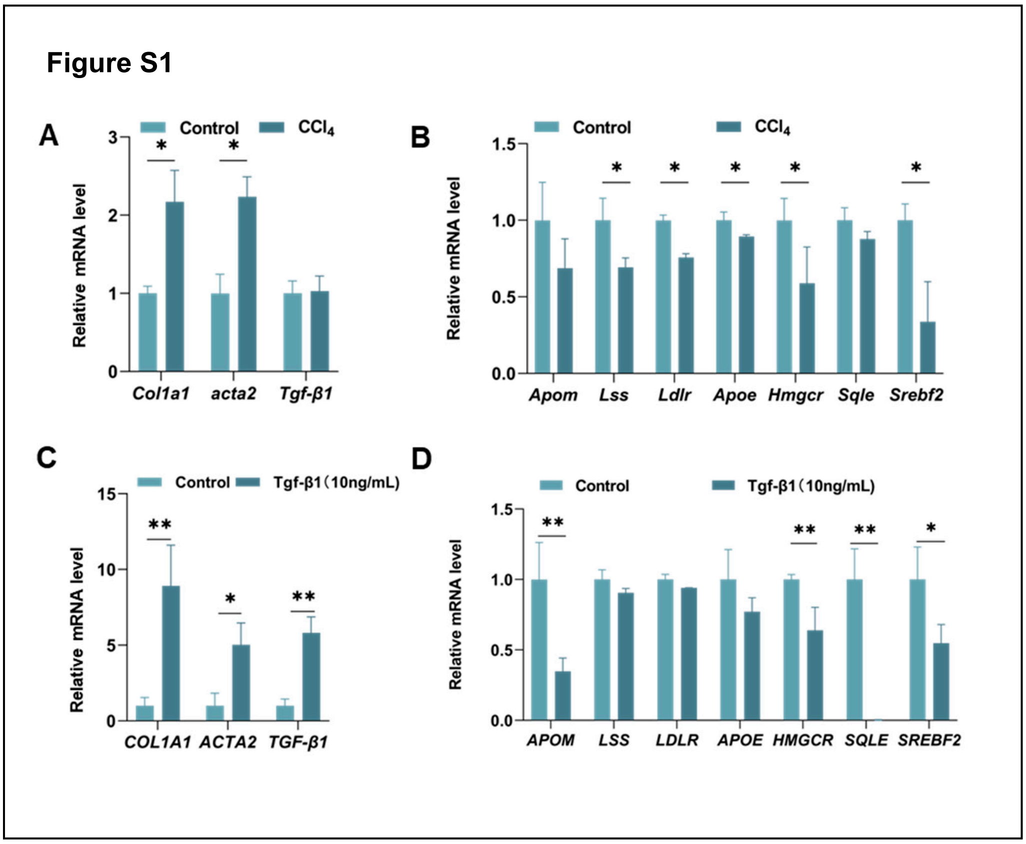

Figure S1. Expression of Cholesterol Metabolism-Related Genes during Liver Fibrosis. (A and B) Relative mRNA levels of fibrosis markers (*Col1a1*, *acta2*, *Tgf-β1*), and genes involved in cholesterol metabolism (*Apom*, *Lss*, *Ldlr*, *Apoe*, *Hmgcr*, *Sqle, Srebf2*) were determined by RT-qPCR in liver tissue of mice treated with corn oil or CCl_4. (C and D) Relative mRNA levels of fibrosis markers (*Col1a1*, *acta2*, *Tgf-β1*) and cholesterol metabolism genes (*APOM*, *LSS*, *LDLR*, *APOE*, *HMGCR*, *SQLE, SREBF2*)were determined by RT-qPCR in LX-2 cells treated with/without TGF-β1 for 24 hours. Statistical significance was analyzed using a two-tailed Student's t test (*p<0.05, **p < 0.01, [n]=3).

Figure S2

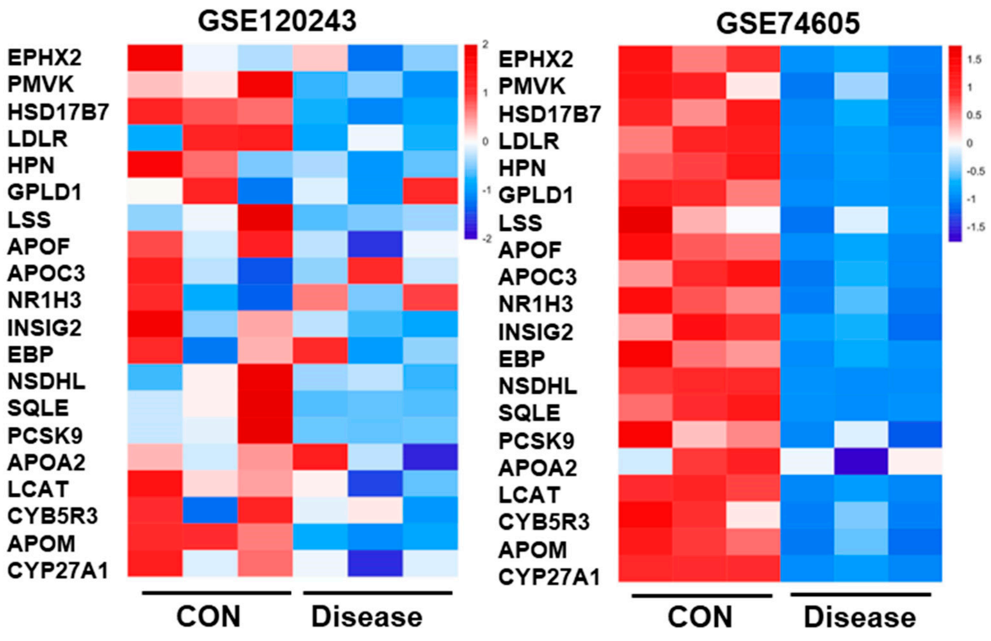

Figure S2. Heatmap of the genes involved in cholesterol metabolism in GEO database. Number of independent experiments, [*n*]=3.

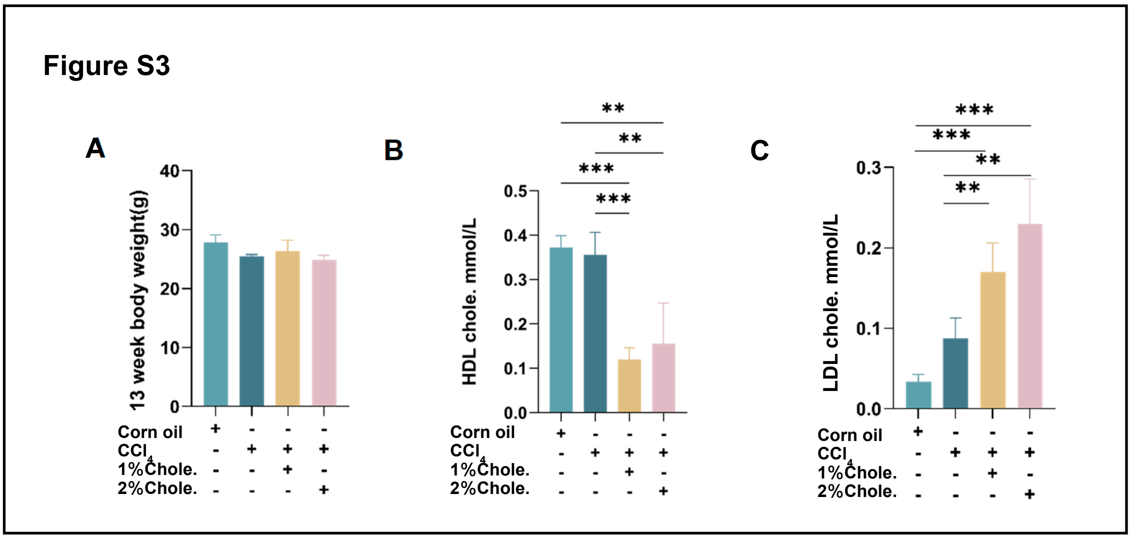

Figure S3. Cholesterol promotes the progression of hepatic fibrosis in mice. (A) Body weight of mice in the 13th week. (B) High density lipoprotein cholesterol (HDL-C) level. (C)Low density lipoprotein cholesterol (LDL-C). *$p < 0.05$, **$p < 0.01$, ***p < 0.001, [n]=3.

Figure S4

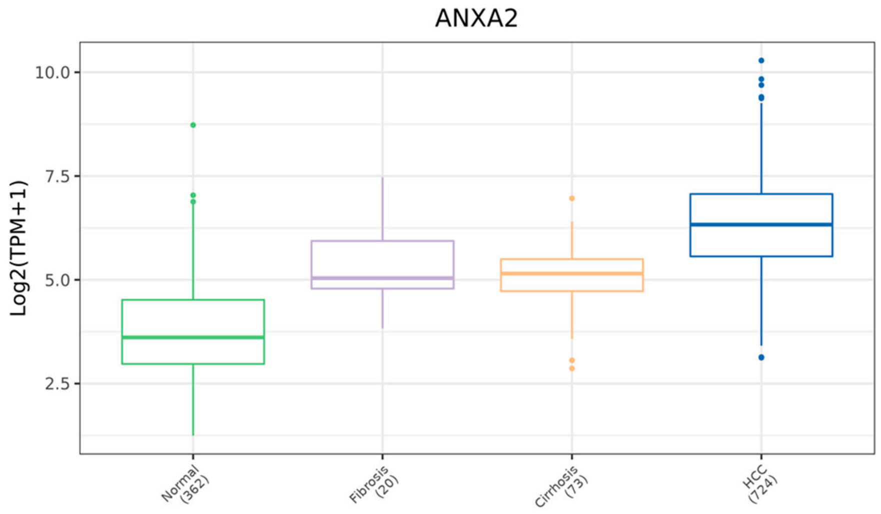

Figure S4. ANXA2 mRNA expression is upregulated during liver disease progression.

Supporting Table S1. Diet Disclosure （1%-2% Cholesterol diet）

Ingredient	AIN-93G Base （control）	1% Cholesterol (AIN-93G)	2% Cholesterol (AIN-93G)
Casein	200	200	200
L-Cystine	3	3	3
Corn Starch	397.5	397.5	397.5
Maltodextrin	132	132	132
Sucrose	100	100	100
Cellulose	50	50	50
Soybean Oil	70	70	70
Vitamin Mix V10037	10	10	10
Mineral Mix S10022G	35	35	35
Cholesterol	-	10.2	20.5
Choline Bitartrate	2.5	2.5	2.5
Total	1000	1010.2	1020.5

Supporting Table S2. The RNA-seq databases information.

GEO no.	Species	Strain	Platforms	Description	PubMed ID
GSE120243	Mus musculus	C57BL/6J	Illumina MouseWG-6 v2.0	C57BL/6J mice were fed a normal diet, high-fat diet (HFD), or high-fat + 0.05% (w/w) UR diet for 16 weeks.	30423963
GSE74605	Mus musculus	C57BL/6	Illumina MouseRef-8 v2.0	Liver inflammation, ductular reaction (DR) and fibrosis were induced in C57BL/6 mice by thioacetamide (TAA) administration for up to 12 weeks.	N/A
GSE130129	Homo sapiens; Mus musculus		Agilent-028005 SurePrint G3 Mouse GE 8x60K; Illumina HiSeq 2500	Including mice, HepG2 cells and primary hepatocytes.	32968110
GSE35961	Mus musculus	C57BL/6	Affymetrix Mouse Genome 430 2.0 Array	Eight-week-old C57BL/6 mice were fed a methionine- and choline-deficient (MCD) + high fat (HF) diet with or without 0.1% metformin for 8 weeks.	23028442
GSE77503	Mus musculus	C57BL/6	Illumina MouseRef-8 v2.0	Total RNA isolated from tumoral and non-tumoral tissue as well as PBS and DDC control tissue.	26844528
GSE99010	Mus musculus	C57BL/6	Illumina HiSeq 2500	Normal diet-fed mice, CCl_4-treated mice, western diet (WD)-fed mice and WD-fed and CCl_4-treated mice at 12 weeks and 24 weeks were examined.	29572095

Supporting Table S3. Primers for Real-Time PCR detection

Primers	Forward (5'→3')	Reverse (5'→3')
hCOL1A1	GAGGGCCAAGACGAAGACATC	CAGATCACGTCATCGCACAAC
hACTA2	AAAAGACAGCTACGTGGGTGA	GCCATGTTCTATCGGGTACTTC
hTGF-β1	GGCCAGATCCTGTCCAAGC	GTGGGTTTCCACCATTAGCAC
hAPOM	GCTACCATCCGCATGAAAGAT	CTGGCCTGTCTCATTCAGCA
hLSS	GCACTGGACGGGTGATTATGG	TCTCTTCTCTGTATCCGGCTG
hLDLR	TCTGCAACATGGCTAGAGACT	TCCAAGCATTCGTTGGTCCC
hAPOE	GTTGCTGGTCACATTCCTGG	GCAGGTAATCCCAAAAGCGAC
hHMGCR	TGATTGACCTTTCCAGAGCAAG	CTAAAATTGCCATTCCACGAGC
hSQLE	GGCATTGCCACTTTCACCTAT	GGCCTGAGAGAATATCCGAGAAG
hSREBP2	CCTGGGAGACATCGACGAGAT	TGAATGACCGTTGCACTGAAG
mCol1α1	GCTCCTCTTAGGGGCCACT	ATTGGGGACCCTTAGGCCAT
mActa2	CCCAGACATCAGGGAGTAATGG	TCTATCGGATACTTCAGCGTCA
mTgf-β1	AGGGCTACCATGCCAACTTC	CCACGTAGTAGACGATGGGC
mApom	GTGGTACTTTATTGCGGGAGC	CCACTTTTCGTGCGGATGGTA
mLss	GTGTCTTGGCTGGGTGATAA	GACACCAACACTGACCCTATC
mLdlr	TCAGACGAACAAGGCTGTCC	CCATCTAGGCAATCTCGGTCTC
mApoe	CTCCCAAGTCACACAAGAACTG	CCAGCTCCTTTTTGTAAGCCTTT
mHmgcr	AGCTTGCCCGAATTGTATGTG	TCTGTTGTGAACCATGTGACTTC
mSqle	ATAAGAAATGCGGGGATGTCAC	ATATCCGAGAAGGCAGCGAAC
mSrebp2	GCAGCAACGGGACCATTCT	CCCCATGACTAAGTCCTTCAACT
mTimp1	CGAGACCACCTTATACCAGCG	ATGACTGGGGTGTAGGCGTA
mCol6a1	CTGCTGCTACAAGCCTGCT	GCACGAAGAATAGATCCACAGGG
mCol3a1	CTGTAACATGGAAACTGGGGAAA	CCATAGCTGAACTGAAAACCACC
mCxcl1	ACTGCACCCAAACCGAAGTC	TGGGGACACCTTTTAGCATCTT
mCd55	ACCCCGGTGCATAGAGAAATC	GGATGACGTACTGTTGTCTTGG
mTlr3	GTGAGATACAACGTAGCTGACTG	TCCTGCATCCAAGATAGCAAGT
mCxcl10	CCAAGTGCTGCCGTCATTTTC	GGCTCGCAGGGATGATTTCAA
mCcl2	TAAAAACCTGGATCGGAACCAAA	GCATTAGCTTCAGATTTACGGGT
mIl-1β	GAAATGCCACCTTTTGACAGTG	TGGATGCTCTCATCAGGACAG
mANXA2	ATGTCTACTGTCCACGAAATCCT	TGACTGACCCGTAGGCACTT

Supporting Table S4. 203 cholesterol-associated fibrotic genes

Number	Gene	Number	Gene	Number	Gene	Number	Gene
1	Cyp3a59	2	Grik5	3	C5ar1	4	Gm16731
5	Cyp2c54	6	H2-K2	7	Bhmt	8	Izumo4
9	ENSMUSG00000120841	10	Abcc3	11	Slc12a9	12	Cd63
13	Cyp2c55	14	Gm12909	15	Oat	16	Col5a3
17	Cyp2c50	18	Col20a1	19	9630013D21Rik	20	Gm10642
21	Sqle	22	mt-Co2	23	Raet1e	24	Aldh1l1
25	Lepr	26	Ip6k2	27	Gm34667	28	mt-Tp
29	ENSMUSG00000120050	30	C730036E19Rik	31	Fads3	32	Jam2
33	Gm10925	34	mt-Cytb	35	Gm8016	36	Ccnd1
37	Lpl	38	Cyp51	39	Gm30784	40	Cyp2u1
41	Hmgcs1	42	mt-Tl2	43	Idi1	44	Chrna4
45	Slco1a4	46	Sult1a1	47	Pigr	48	Sult5a1
49	Cyp2c29	50	Ldha	51	Gm11843	52	mt-Tr
53	Hmgcr	54	mt-Atp6	55	Cd36	56	Capg
57	Abcg5	58	mt-Nd6	59	Gm43611	60	Lgi2
61	Apoa4	62	Cyp1a2	63	Gm3839	64	Rps2-ps10
65	Cyp3a25	66	mt-Ts2	67	Igf2bp2	68	Crtap
69	Gstt3	70	mt-Co1	71	Sall1	72	Rcn3
73	Gm28439	74	mt-Nd2	75	Tlcd1	76	Fam214a
77	Msmo1	78	Plin2	79	Cyp2j9	80	App
81	Acacb	82	Mvd	83	Erbb3	84	Vnn1
85	Slc13a2	86	mt-Nd4	87	Slc8a1	88	2210039B01Rik
89	Cyp3a11	90	Cth	91	Atg16l2	92	Cda
93	Cyp26a1	94	Blm	95	mt-Rnr1	96	Acss3
97	Acss2	98	mt-Nd5	99	Ugt1a5	100	Nat8
101	Gramd1b	102	Lpin2	103	Prlr	104	Lrit1

Continued Supporting Table S4. 203 cholesterol-associated fibrotic genes

Number	Gene	Number	Gene	Number	Gene	Number	Gene
105	Fabp2	106	Gtpbp4-ps1	107	Fgf21	108	Ccl6
109	Abcg8	110	Tm7sf2	111	Ctss	112	Gm28437
113	Cyp2b10	114	Lmntd2	115	Hmox1	116	Rdh16
117	9130409I23Rik	118	4930404H11Rik	119	Ednrb	120	Dio1
121	Gm29216	122	Gm49410	123	Mettl27	124	mt-Te
125	Upp2	126	Slc47a1	127	Ces1g	128	Arhgap6
129	Cyp2c37	130	Gm10222	131	Gpd2	132	Manf
133	Orm3	134	Nsdhl	135	BC024386	136	Osbpl3
137	Mfsd2a	138	mt-Atp8	139	Vmn2r3	140	C3ar1
141	Slc15a2	142	Cyp27a1	143	Pdia6	144	Bhlha15
145	Fdft1	146	Sema4g	147	Dpt	148	Anxa2
149	Gm49012	150	ENSMUSG00000120572	151	ENSMUSG00000119959	152	Treml4
153	Adra1b	154	Rdh16f2	155	Kifc2	156	Slc37a2
157	Rpl30	158	Ttbk1	159	Itm2c	160	Gm48600
161	Ces2a	162	H2-Q1	163	Sparc	164	Ccl2
165	Fads2	166	mt-Nd1	167	Colgalt2	168	Rcn1
169	Slc12a7	170	Gm3336	171	Abcg1	172	Gm43936
173	Selenop	174	Fth1	175	U6	176	Inhbb
177	Fam13a	178	Tmem86a	179	C1qb	180	Susd4
181	Akr1d1	182	Meg3	183	Zbtb45	184	Rian
185	Fdps	186	Raet1d	187	Lgr5	188	Tagln2
189	Gm28661	190	Cyp21a2-ps	191	Odf3b	192	Lrit2
193	mt-Th	194	Sdf2l1	195	Gm26608	196	Asf1b
197	Ccne1	198	Lpin1	199	Cox5a	200	Slc15a3
201	ENSMUSG00000120149	202	Rdh11	203	Odad3		

Supporting Table S5. Antibodies

Antibodies	Source	Cat. No.
α-SMA	Proteintech	14395-1-AP
GAPDH	Proteintech	60004-1-Ig

Supporting Table S6. Summary of RNA-seq library quality metrics and genome alignment statistics

Sample	Group	Raw reads	Clean reads	Error rate (%)	Q20 (%)	Q30 (%)	GC (%)	Total mapping (%)	Unique mapping (%)
Con-1	control	42629706	41417452	0.01	98.85	96.63	45.47	88.31	72.54
Con-2	control	46641000	45153078	0.01	98.87	96.65	43.17	94.93	69.35
Con-3	control	46176920	44231934	0.01	98.83	96.59	43.76	93.43	70.44
CCl_4-1	CCl_4	40506824	40438920	0.01	98.77	96.40	45.08	93.36	77.64
CCl_4-2	CCl_4	45296862	44406182	0.01	98.79	96.50	45.78	94.50	82.41
CCl_4-3	CCl_4	46650566	45703630	0.01	98.79	96.58	44.73	94.61	78.23
1%-1	1%Chole.	44532250	43682658	0.01	98.75	96.38	47.36	94.37	85.15
1%-2	1%Chole.	41501722	40713106	0.01	98.79	96.54	46.15	94.10	83.73
1%-3	1%Chole.	45491956	44523808	0.01	98.80	96.54	47.26	94.03	84.69
2%-1	2%Chole.	45396624	44402050	0.01	98.73	96.33	46.70	94.37	83.17
2%-2	2%Chole.	45881882	44596934	0.01	98.81	96.54	47.38	94.13	84.80
2%-3	2%Chole.	40826842	39644990	0.01	98.85	96.61	48.01	95.13	86.89
